# Enhanced Multi-Scale Defect Detection in Steel Surfaces via Innovative Deep Learning Architecture

**DOI:** 10.3390/s26062001

**Published:** 2026-03-23

**Authors:** Zhaoxuan Zhou, Yan Cao

**Affiliations:** 1 School of Mechanical and Electrical Engineering, Xi’an Technological University, Xi’an 710021, China; zhouzhaoxuan@st.xatu.edu.cn; 2 School of Computer Science and Engineering, Xi’an Technological University, Xi’an 710021, China

**Keywords:** steel surface defects, object detection, CBY parallel network structure, TFF-PANet, deep learning

## Abstract

**Highlights:**

**What are the main findings?**

- A CBY parallel backbone module is proposed in the feature extraction stage to enhance the network’s ability to understand input data and improve its fine-grained feature extraction capability for minor defects.
- In the feature fusion network, a novel TFF-PANet feature extraction architecture is designed to optimize multi-scale feature interaction, enhance heterogeneous feature fusion, and improve detail-capturing capabilities.
- The GS-Head (Grained Structure Head) detection head is designed for deployment on edge devices. This design preserves feature channel connections, enhances image texture, and compresses the model.

**What is the implication of the main finding?**

- The proposed CBY parallel backbone module improves the model’s performance in recognizing fine-grained defects, making it more efficient in defect detection.
- The TFF-PANet architecture optimizes multi-scale feature interaction and enhances feature fusion, improving the model’s ability to capture detailed and diverse features.
- The GS-Head design ensures that the model is lightweight enough for deployment on edge devices, while still preserving critical feature information and enhancing image texture.
- The algorithm’s superior performance on the NEU-DET and GC10-DET datasets highlights its robustness and generalization capability, making it an effective solution for real-world industrial defect detection applications.

**Abstract:**

Steel surface defects significantly impact product quality and safety in industrial settings. Traditional defect detection methods suffer from inefficiencies and limitations. This study introduces an innovative deep learning architecture, CTG-YOLO, designed to enhance multi-scale defect detection accuracy on steel surfaces. By integrating a CBY parallel network structure, a TFF-PANet neck network, and a GS-Head detection head, our model achieves superior feature extraction and fusion capabilities. Experimental results on the NEU-DET and GC10-DET datasets demonstrate significant improvements, with mean Average Precision (mAP) scores of 76.55% and 69.94%, respectively, outperforming the original YOLOv8s by 3.72% and 3.14%. This research provides a robust foundation for industrial defect detection applications.

## 1. Introduction

**Figure 1:**
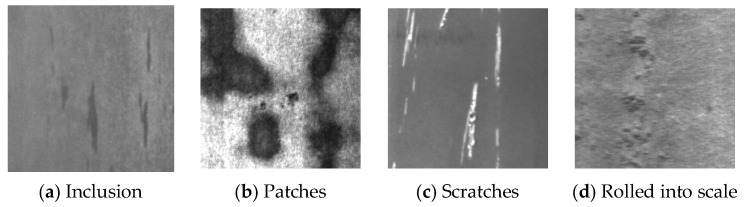
Common surface defects of steel.

Steel, as one of the most fundamental manufacturing materials, is widely used in defense, automotive, aerospace, and consumer goods industries due to its excellent mechanical properties, superior surface quality, and relatively low cost. However, during production and processing, steel surfaces can develop various defects (as shown in Figure 1a–d) due to factors such as raw material limitations, production methods, equipment constraints, and environmental conditions [1]. These defects not only compromise product aesthetics but also reduce service life and may even pose safety risks in industrial applications. Therefore, surface quality inspection of steel holds significant research importance. Currently, the most widely used defect detection methods include manual visual inspection, eddy current testing, magnetic flux leakage testing, infrared testing, and ultrasonic testing [2]. However, manual visual inspection [3] suffers from low efficiency, strong subjectivity, and difficulty in identifying micro-defects. Eddy current testing and magnetic flux leakage testing are material-dependent and struggle with quantitative analysis. Infrared testing and ultrasonic testing require highly skilled operators, are sensitive to environmental conditions, have limited sensitivity, and demand advanced equipment.

Compared with traditional defect detection methods, deep learning-based defect detection methods have gradually attracted the attention of modern enterprises due to their excellent performance and high detection efficiency. Currently, there are three mainstream frameworks for deep learning-based detection models: two-stage detection frameworks, one-stage detection frameworks, and Transformer-based detection frameworks. Representative models of the two-stage detection framework include R-CNN [4], Faster R-CNN [5], and Mask R-CNN [6]. The principle of this type of algorithm is to first generate candidate regions, then classify and regress the candidate bounding-box regions. Therefore, such algorithms high detection accuracy but suffer from drawbacks such as considerable computational complexity, a large number of parameters, and slow detection speed. Representative models of the one-stage detection framework include YOLO (You Only Look Once) [7] and SSD (Single-Shot MultiBox Detector) [8]. The principle of this type of algorithm is to directly predict the categories and positions of targets based on regression algorithms, achieving faster detection speeds and making them suitable for real-time detection applications. A representative model of the Transformer-based detection framework is DETR (DEtection TRansformer) [9]. The principle of this algorithm is to utilize the Transformer architecture for object detection, capturing global information through a self-attention mechanism. It is characterized by high detection accuracy but long training times and considerable computational complexity.

In this paper, an optimized model called CTG-YOLO is proposed for steel defects based on YOLOv8. First, a novel parallel CBY module is designed in the backbone network to enhance the network’s ability to understand data. Secondly, at the feature fusion stage, a brand-new neck structure named TFF-PANet (Triple Feature Fusion PANet) is designed to achieve multi-level feature fusion, capture detailed information at different scales, and strengthen the feature expression ability. Finally, at the prediction stage, a GS-Head detection head is designed to enhance the textural information of the image, compress the model size, and simultaneously improve the model’s performance. The main contributions of this work are summarized as follows:

(1) In response to the challenges in detecting surface defects on steel materials, a novel parallel backbone module named CBY (composed of a ConvNext block and C2f module) is designed at the feature extraction stage of the backbone network. This module aims to address the limitations in detecting small targets, enhance the network’s understanding of input data, and efficiently extract detailed information of tiny defects.
(2) In the feature fusion network, a novel feature extraction architecture named TFF-PANet is meticulously designed to enhance the ability to capture fine details and optimize the interaction and fusion among heterogeneous features. By facilitating interactions between feature maps of different scales, this architecture significantly improves the model’s ability to capture detailed information, strengthens the integration of global contextual features, and ultimately enhances the multi-scale feature representation capacity.
(3) To facilitate the deployment of the model on edge devices, a GS-Head (Grained Structure Head) detection head is designed. This head preserves the information connections between feature channels to the greatest extent and enhances the textural information of the image while simultaneously achieving model compression.
(4) Experiments conducted on the NEU-DET and GC10-DET datasets demonstrate the effectiveness of the proposed algorithm compared with mainstream object detection methods. The results validate its superior robustness and generalization capabilities in steel surface defect detection tasks.

The remaining sections of this paper are arranged as follows: Section 2 introduces the related work, especially the latest defect detection methods. Section 3 elaborates on the structure and principle of the CTG-YOLO model proposed in this paper. In Section 4, experiments and performance comparisons are carried out to verify the effectiveness of the model. Finally, in Section 5, the conclusions are presented.

## 2. Related Work

This part mainly introduces the technologies related to this study, including object detection based on traditional methods and object detection based on deep learning.

### 2.1. Traditional Object Detection

Traditional object detection algorithms have been applied to defect detection tasks for a long time and have been further optimized in this field. Traditional object detection algorithms mainly rely on manually extracted features or machine learning for object recognition and localization, e.g., Support Vector Machine (SVM) [10], decision trees [11], AdaBoost [12], etc. For example, Zhang et al. [13] optimized a detection system, selected kernel functions, and set parameters in the SVM method, enabling the SVM method to effectively identify seven types of defects. Agarwal et al. [14] proposed a process knowledge-based support vector classification scheme, PK-MSVM, which combines the feature extraction task of automatic detection with process knowledge, greatly improving detection reliability. Pernkopf et al. [15] established the structure of a Bayesian network and applied a floating search algorithm, achieving a good balance between the classification performance of structure learning and computational efficiency. Traditional object detection methods rely on manually extracted features, and the algorithmic structure is relatively complex. The detection performance often fails to meet the requirements of industrial production, and due to their limitations, such methods are gradually being replaced by deep learning-based methods.

### 2.2. Deep Learning-Based Object Detection Algorithms

With the development of deep learning technology, due to its characteristics of high efficiency, accuracy, and flexibility, it has been widely applied to the field of steel defect detection. Generally, object detection algorithms can be divided into two major categories: two-stage and one-stage methods. Two-stage algorithms have higher accuracy but are slower, while one-stage algorithms are faster but have slightly lower accuracy. For example, Li et al. [16] improved Faster R-CNN and Feature Pyramid Network (FPN) and adopted multi-scale feature fusion to achieve higher accuracy in steel strip detection. Wang et al. [17] proposed the combination of the improved ResNet50 with Faster R-CNN to reduce the running time and improve accuracy. Yin et al. [18] introduced a feature pyramid into the Faster R-CNN network, enabling the network to combine high-level and low-level feature information, greatly improving the detection accuracy. Tang Maojun et al. [19] enhanced spatial semantic information by introducing the FPN structure and the decoupled classification refinement structure and proposed a Faster R-CNN defect detection algorithm with the classification network and the localization network placed in parallel. The advantage of two-stage detection algorithms lies in their high-precision object detection ability. However, they also face the challenges of considerable computational complexity and poor real-time performance, which restrict their application in industrial production. Therefore, researchers have proposed one-stage object detection algorithms to improve the detection speed.

One-stage object detection algorithms directly perform object classification and localization on images to achieve end-to-end operations. Zhao et al. [20] proposed a steel surface defect detection model that improves YOLOv5. By designing the Res2Net module to expand the receptive field and the dual-feature pyramid network to enrich semantic information, it achieves good detection performance. Li et al. [21] proposed an improved YOLOv7 algorithm. They added feedback connections in the feature fusion stage, designed a detection head based on the collaborative attention mechanism in the prediction stage, and applied it to circuit-board defect detection. Lu et al. [22] proposed an improved YOLOv8 algorithm. The design’s dynamic convolution to optimize the C2f module and the adoption of a dynamic, non-monotonic focusing mechanism improved the overall performance of the steel surface detector. Liao et al. [23] proposed an improved YOLOv8 algorithm for potato defect detection, introducing the lightweight HGNetv2 network to enhance the feature extraction ability, designing a cross-scale feature fusion CCFM module to optimize network performance, and using the structural reparameterization strategy to enhance the detection performance and improve the computational efficiency. Generally speaking, although one-stage object detection algorithms have relatively poor detection accuracy, they can achieve simultaneous improvement of detection speed and accuracy by optimizing their internal modules and inserting attention modules. Through research on the basic YOLOv8 model, we proposed the CTG-YOLO model to enhance the detection of small and medium-sized defects on steel surfaces and improve the overall detection accuracy.

## 3. Methods

### 3.1. Baseline Networks

**Figure 2:**
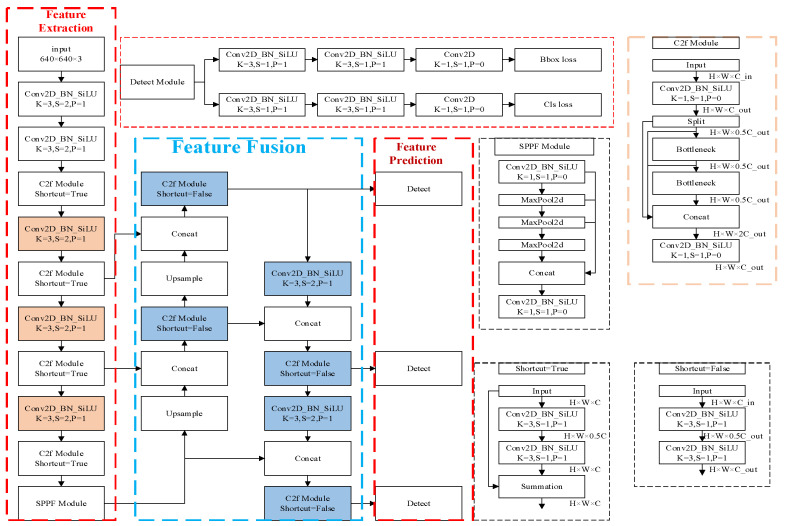
The network structure of YOLOv8.

YOLOv8 is an efficient real-time object detection model developed by Ultralytics. Its architecture is shown in Figure 2. While maintaining high detection accuracy, it achieves a relatively fast detection speed and is often used for real-time object detection on resource-constrained devices. The network architecture of YOLOv8 mainly consists of three parts: the backbone, the neck, and the head. The backbone network uses the CSPDarkNet structure composed of multiple convolutional layers, C2f modules, and a Spatial Pyramid Pooling layer (SPPF) for feature extraction. The neck network adopts the PAN-FPN structure to achieve multi-scale feature fusion and feature expression. The head network is responsible for the final object detection and classification tasks. The YOLOv8 model includes YOLOv8n, YOLOv8s, YOLOv8m, YOLOv8l, and YOLOv8x, with their sizes ranging from small to large, and they are suitable for different application scenarios. Just like the optimized algorithm proposed in this paper, due to the need for detection accuracy and speed when applied to mobile devices, this paper will optimize and design a high-precision network model based on YOLOv8s.

### 3.2. The Improved CTG-YOLO

**Figure 3:**
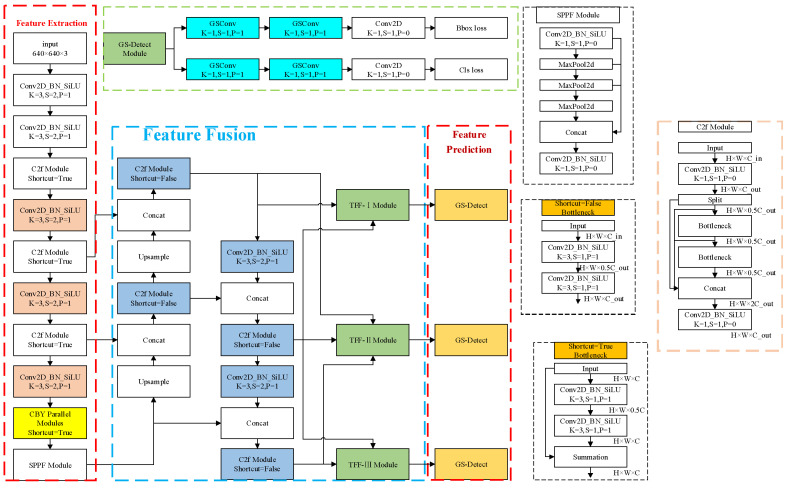
The network structure of CTG-YOLO.

To improve the detection accuracy of small and relatively blurry defects on steel surfaces, this paper proposes the CTG-YOLO method, with the network architecture shown in Figure 3. First, the steel surface defect image is input into the CBY module, which is composed of parallel ConvNext and C2f modules, to enhance the network’s ability to understand the data. Then, the multi-level feature maps generated by the backbone network are input into the TFF-PANet module of the neck network, where high-level semantic information and low-level detail information from different feature layers are fully fused, enhancing the expression capability of multi-scale features. Finally, the feature maps of different scales generated by the neck network are input into the decoupled prediction layer composed of GSconv, which maximizes the retention of information correlation between feature channels, enabling the network to accurately locate and classify defects.

#### 3.2.1. The Enhancement of the Backbone Network

To address the issue of diverse shapes of steel surface defects and their similarity to the background, this paper designs the CBY parallel network module in the feature extraction stage. The module fuses the lightweight ConvNext Block [24] and C2f module in parallel, which expands the receptive field while enhancing the ability to extract detailed information, achieving complementary and fusion of multi-scale features and improving the model’s ability to capture contextual information.

**Figure 4:**
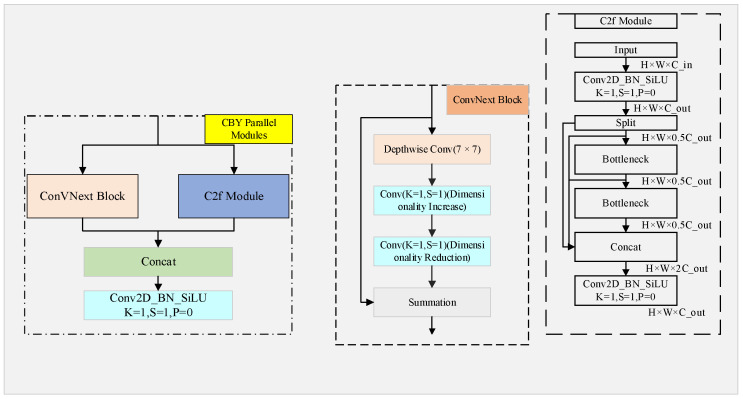
The CBY parallel network model.

The structure of the CBY module is shown in Figure 4. First, it extracts rich features through a dual-branch parallel structure. Then, the information from both branches is fused through a concatenation operation to enhance the network’s understanding of the data. Finally, a 1 × 1 convolution is applied to adjust the number of channels, enabling information interaction between channels and enhancing nonlinear features.

Compared with the single-branch C2f module, the CBY module significantly improves the stability and representational capability of feature extraction, effectively solving the challenge of multi-scale feature extraction in defect detection.

#### 3.2.2. The Enhancement of the Neck Network

In defect detection, traditional multi-scale feature fusion modules typically integrate features from adjacent layers through simple upsampling or downsampling, which makes it difficult to fully fuse features at different resolutions. To effectively combine the detailed information from large-scale feature layers with the high-level semantic information from small-scale feature layers, this paper proposes the TFF-PANet module. To address the issue of the neck network having only a single-scale input and lacking cross-scale feature interaction, we introduce a multi-scale feature-splitting and fusion mechanism based on PANet, aiming to enhance the information richness of each feature layer and improve fusion effectiveness.

**Figure 5:**
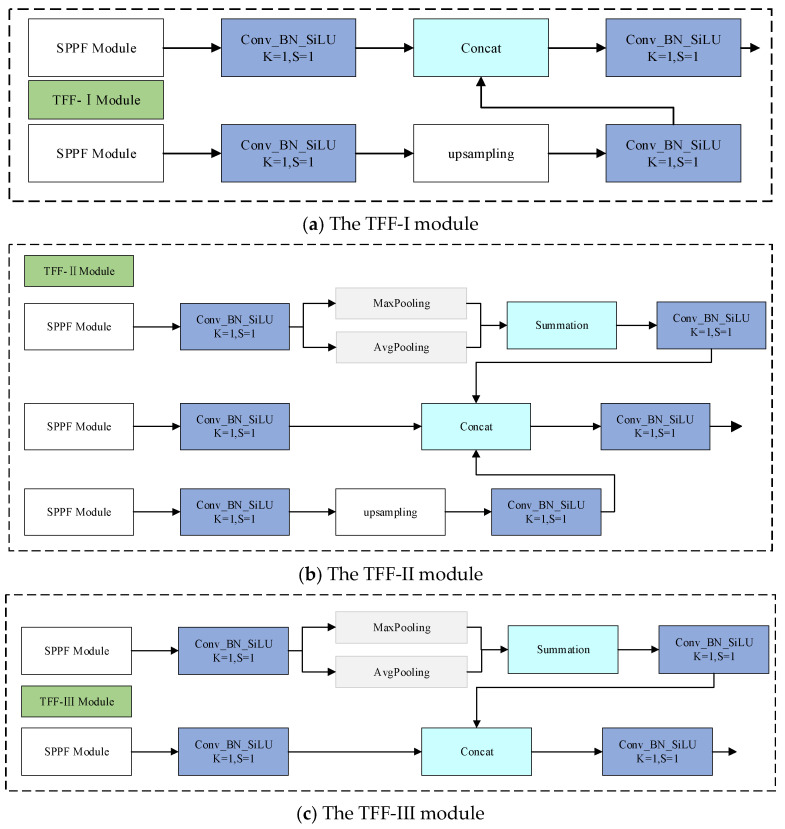
The TFF modules at different scales.

The neck module is used to process multi-scale features extracted by the backbone network (as shown in Figure 5a–c). Its processing flow consists of two stages: First, the smaller-scale feature maps are upsampled, and feature fusion is achieved through lateral connections with adjacent feature layers; then, the larger-scale feature maps are downsampled, and the fusion effect is further enhanced through lateral connections. Finally, feature maps of three scales—20 × 20, 40 × 40, and 80 × 80—are output. However, while the above fusion strategy strengthens feature representation, the issue of feature information loss becomes more severe as the network deepens, and the quality of information is not fully addressed. To tackle this issue, this paper proposes the TFF-PANet module based on PANet. The module first uses convolution operations to unify the channel number of the three output feature maps. Then, parallel max pooling and average pooling are applied to the large-scale feature maps for downsampling, enhancing the network’s adaptability to spatial variations. The smaller-scale feature maps are upsampled, and adjacent pixel padding is applied. Finally, the three processed feature maps with the same dimensions are convolved and concatenated along the channel dimension.

Through this multi-scale feature aggregation mechanism, TFF-PANet effectively fuses the detailed information of large-scale feature maps with the high-level semantic information of small-scale feature maps into the main scale feature map, significantly enhancing feature reuse and fusion capability and effectively mitigating the issue of information loss in deep networks.

#### 3.2.3. Head Network

**Figure 6:**
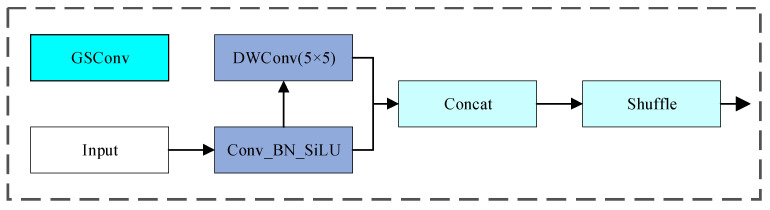
Structural diagram of GSConv.

**Figure 7:**
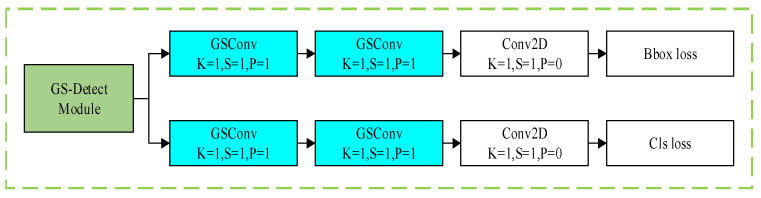
The GS-Detect detection head.

The head network is the prediction layer of the detection model and is mainly responsible for converting the features extracted by the previous network into final prediction results. Specifically, the model uses a decoupled head to separately extract the target location and class information and processes them independently through different branches, enabling the model to more accurately predict the classes of pixels when dealing with images. Although the use of the decoupled head greatly improves the prediction accuracy, the construction using 3 × 3 standard convolution makes the model generate a large number of parameters and results in considerable computational complexity [25]. Therefore, to ensure the accuracy of the model and reduce the computational complexity of the detection layer, this paper designs a GS-Head detection head, replacing the standard convolution with GSConv [26], with its structure shown in Figure 6. It retains the information connection between feature channels, enhances the textural information of the image, and compresses the model size. As shown in Figure 7, the main idea of GSConv is to uniformly exchange feature information on different channels through a shuffle operation so that the information of depthwise separable convolution and the information of standard convolution are mixed and output, improving the detection performance of the network.

## 4. Experiment

### 4.1. Datasets

**Figure 8:**
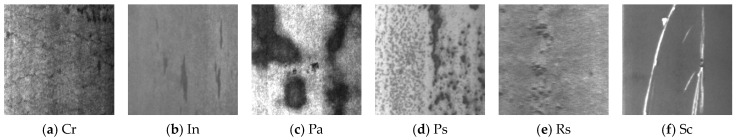
NEU-DET image samples.

In this paper, the NEU-DET dataset [27] and the GC10-DET dataset [28] are used for training and validation to prove the effectiveness of the improved model. The NEU-DET dataset, produced by the team of Song Kechen from Northeastern University, covers six common types of surface defects of hot-rolled steel strips—namely, crazing (Cr), inclusions (In), patches (Pa), pitting (Ps), rolled-in scale (Rs) and scratches (Sc). Each type of defect has 300 images, and the sample defect images are shown in Figure 8a–f. The GC10-DET dataset is a surface defect dataset collected in real industry that contains 2294 defect images and ten types of surface defects of steel plates—namely, punching (Pu), weld (Wi), crescent gap (Cg), water stain (Ws), oil stain (Os), silk stain (Ss), inclusion (In), roll pit (Rp), crease (Cr) and waist crease (Wf). In this experiment, for the training set, test set and validation set, a random division of 8:1:1 is adopted. The used datasets are public and rich in variety, including defect images taken from different angles and in different environments.

### 4.2. Experimental Environment and Model Configuration

The experiments and model performance tests in this study were conducted on a Windows 11 operating system. The experimental environment was configured as follows: an RTX 3080 Ti GPU with 12 GB of VRAM, a 12-core vCPU Intel(R) Xeon(R) Silver 4214R CPU @ 2.40 GHz, 30 GB of RAM, and CUDA version 11.0. The model parameters were set as follows: input image size of 640 × 640 pixels, batch size of 8, and 200 training epochs (including 50 frozen epochs to improve training efficiency). The SGD optimizer was used, with a momentum of 0.937 and a weight decay of 0.0005 to prevent overfitting. The initial learning rate was 0.01, which was dynamically adjusted using a cosine annealing schedule. To ensure fairness in the comparative experiments, both the ablation studies and model training utilized pre-trained weights from the VOC2007 + 2012 public datasets. The specific parameters are shown in Table 1.

The performance metrics used to evaluate the modified algorithm mainly include Mean Average Precision (mAP), model parameters (Params), and the floating-point operations (FLOPs) that represent the model’s complexity. These metrics are used to objectively assess the performance of the modified network.

Mean Average Precision (mAP) is based on the precision (P) and recall (R) to form a curve and the area under the P–R curve. A larger area under the curve indicates higher accuracy in category detection. The evaluation parameters are shown in Table 2. Some of the formulae for these metrics are presented in Equations (1)–(3).

(1) $$P=\frac{TP}{TP+FP}$$

(2) $$R=\frac{TP}{TP+FN}$$

(3) $$mAP=\frac{1}{n}\sum_{i=1}^{n}\int_{0}^{1}P\left(R\right)dR$$

Among them, n is the number of sample categories to be detected.

Furthermore, the evaluation of the proposed model considers important indicators such as parameter count, computational complexity, and detection speed. These metrics provide a comprehensive assessment of the algorithm’s accuracy, recall rate, adaptability, and efficiency, serving as crucial evaluation metrics for improved defect detection performance.

### 4.3. The Comparative Experiment on the NEU-DET Dataset

In order to verify the feasibility of the CTG-YOLO model in industrial settings, this study selects some representative models for comparative experiments. The representative models include YOLO-series algorithms (such as YOLOv3-tiny, YOLOv4, YOLOv7-tiny, YOLOv8s, YOLOv9s, and YOLOv10s), Faster R-CNN, SSD, and other improved models. The performance indicators on the NEU-DET dataset are summarized in Table 2. Through comparison with these models, the effectiveness and superiority of CTG-YOLO can be further verified.

Table 2 comprehensively compares the detection accuracy, model complexity, and real-time performance of typical object detection models on the NEU-DET dataset. Experimental results show that the proposed algorithm achieves significant advantages in detecting crazing (Cr) and pitted surface (Ps) defects, with AP values reaching 42.09% and 90.35%, respectively, corresponding to improvements of 6.51% and 2.38% over the baseline YOLOv8s. Particularly for the low-contrast, complex-texture crazing defects, CTG-YOLO efficiently extracts fine-grained features through the CBY parallel structure and enhances global feature fusion via the TFF module, effectively improving detection accuracy and achieving a substantial breakthrough.

In terms of computational complexity, two-stage detection algorithms such as Faster-RCNN have significant shortcomings. Their numbers of FLOPs reach as high as 402.02 G, and FPS is only 20.7, resulting in high resource consumption and poor real-time performance, making them unsuitable for high-speed online detection in production lines. Lightweight models like YOLOv3-tiny and YOLOv7-tiny, while having relatively low number of FLOPs of 12.9 G and 13.8 G respectively and high FPS, suffer from a significant drop in detection accuracy, with mAP values of only 68.6% and 66.4%, failing to meet the accuracy requirements for steel surface defect detection. Models like YOLOv9s and FCOS, which are popular in single-stage detection, have improved accuracy, but YOLOv9s has increased model complexity, resulting in 38.7 G FLOPs, which causes a noticeable drop in real-time performance (FPS of 31). FCOS has a higher computational complexity, with 50 G FLOPs and an mAP lower than that of CTG-YOLO.

**Figure 9:**
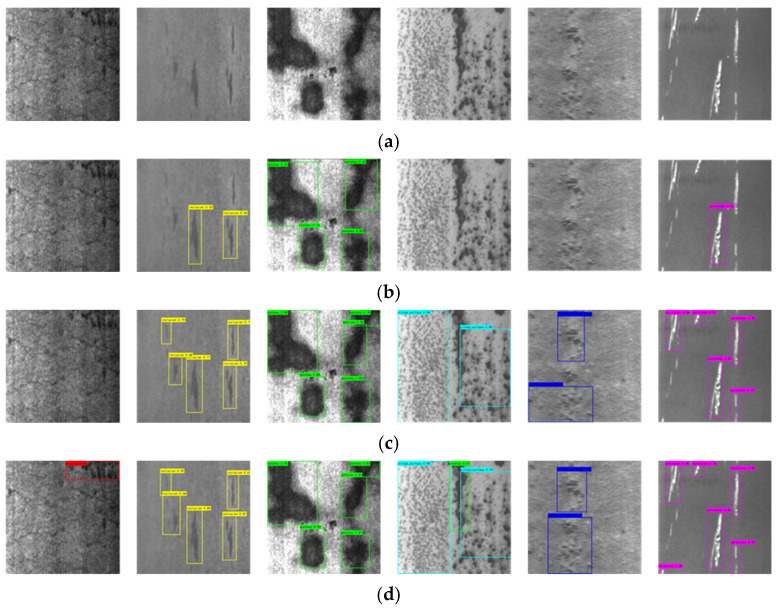
Visualization of the detection results of different algorithms on the NEU-DET dataset. (**a**) Original images of different defect detections. (**b**) Visualization of the detection results of YOLOv7-tiny on the NEU-DET dataset. (**c**) Visualization of the detection results of YOLOv8s on the NEU-DET dataset. (**d**) Visualization of the detection results of CTG-YOLO on the NEU-DET dataset.

In contrast, CTG-YOLO strikes a good balance between detection accuracy, computational complexity, and real-time performance. It achieves the highest mAP of 76.55%, outperforming all other compared models. It has fewer FLOPs (27.216 G) than the baseline YOLOv8s model (28.817 G) and most other mainstream models, resulting in lower computational costs. The FPS is 122, which is slightly higher than YOLOv8s, far exceeding YOLOv9s, Faster-RCNN, and other models and meeting the real-time detection requirements in industrial scenarios. Although the parameter count of 13.667 M is slightly higher than that of YOLOv8s (11.167 M), the significant improvement in detection accuracy and the optimized computational complexity make this increment reasonable for engineering applications. Additionally, CTG-YOLO maintains excellent detection accuracy for inclusions (In) and rolled-in scale (Rs) defects, making it suitable for practical engineering applications. Visualization results (Figure 9a–d) show that the proposed algorithm improves detection accuracy compared to other models, effectively detecting various steel surface defects with lower computational costs and high real-time performance.

### 4.4. Comparative Experiment on the GC10-DET Dataset

**Figure 10:**
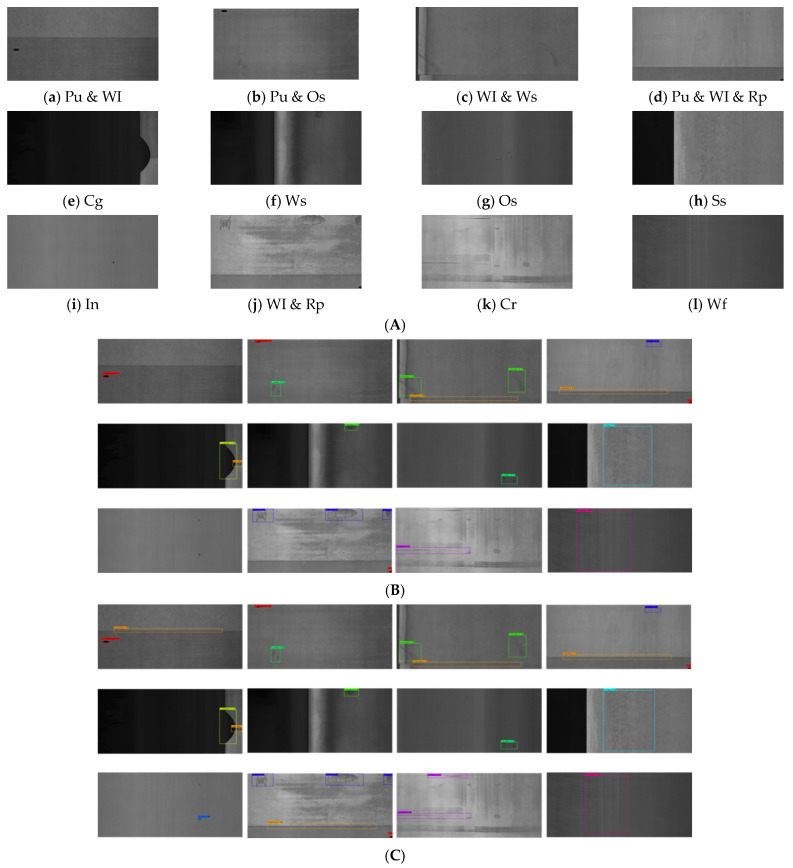
The detection results of YOLOv8s and CTG-YOLO on the GC10-DET dataset. (**A**) Typical steel surface defects in the GC10-DET dataset. (**B**) Visualization of the detection results of the YOLOv8s algorithm on the GC10-DET dataset. (**C**) Visualization of the detection results of the CTG-YOLO algorithm on the GC10-DET dataset.

In order to further explore the effectiveness of CTG-YOLO, the detection performance test was carried out on the GC10-DET dataset, the results of which are shown in Figure 10A–C. Compared with the NEU-DET steel surface defect dataset released by Northeastern University, the GC10-DET dataset covers a wider range of defect types and is closer to changeable industrial scenarios. The defect categories are shown in the Figure 10(Aa–Al). For comparison, the detection algorithms used on the GC10-DET dataset were mainly selected. However, considering that some models do not have verification results on the GC10-DET dataset, some improved algorithms with excellent performance are also introduced for comparison. In the experiment, AP and mAP were used as evaluation indicators.

The comparison results are shown in Table 3. Analysis of specific defect types shows that CTG-YOLO demonstrates excellent performance in the detection of inclusions (In) and creases (Cr), achieving AP values of 45.79% and 52.38%, which are 10.49% and 11.48% higher than those of the FPDNet algorithm. This indicates that CTG-YOLO enhances the multi-scale feature expression ability of the model. For the detection of punched holes (Pu), welds (Wl), crescent-shaped gaps (Cg), water spots (Ws), oil spots (Os), silk spots (Ss) and waist creases (Wf), although there are certain fluctuations, satisfactory results were also achieved. Overall, CTG-YOLO shows excellent comprehensive capabilities, proving the effectiveness and generalization ability of the improved model and providing strong evidence for the practicality of the improved module.

Although the mAP of CTG-YOLO exceeds that of most algorithms and its detection result for inclusions (In) also reached an astonishing 42.45%, unfortunately, CTG-YOLO seems to continue the common problem of YOLO-series algorithms. In the detection of rolled pits (Rp), its detection accuracy is only 20%. This is because such defects are quite similar to the background, and they are easily treated as the background during detection, posing a detection challenge for this type of defect in the model. Although CTG-YOLO enhanced the multi-scale expression ability of the model by introducing the CBY and TFF modules, it offers little help in the field of image information contrast. Liu [35] enhanced image contrast information through edge enhancement at the graphic input stage, and the detection accuracy of rolled (Rp) defects reached an astonishing 48.8%. However, the detection accuracy of other defect types decreased to a certain extent, demonstrating the disadvantages of edge enhancement for the improved algorithm. Therefore, the next step of work is to enhance the fine-grained geometric information in the image while ensuring the improvement of the model’s detection accuracy, providing substantial help for the detection of difficult-to-detect defects such as rolled (Rp) defects.

### 4.5. Ablation Experiment

In order to further demonstrate the influence of each improved module on the model, this paper conducts ablation experiments on the NEU-DET dataset with YOLOv8s as the base model to prove the applicability of each algorithm. In order to accurately evaluate the performance and complexity of the model, AP, mAP, FLOPs, FPS and the number of parameters are used as evaluation indicators for the ablation experiment. In order to more intuitively display the ablation results, in this paper, different improvement methods are abbreviated as follows:

- Baseline.
- The combination of Baseline and ConvNext-C2f parallel structure is abbreviated as C-YOLO.
- The combination of Baseline and the TFF-PANet neck structure is abbreviated as T-YOLO.
- The combination of Baseline and the GSconv prediction head is abbreviated as G-YOLO.
- The combination of Baseline, ConvNext-C2f and TFF-PANet is abbreviated as CT-YOLO.
- The combination of Baseline, ConvNext-C2f and GSconv is abbreviated as CG-YOLO.
- The combination of Baseline, TFF-PANet and GSconv is abbreviated as TG-YOLO.
- The combination of Baseline, ConvNext-C2f, TFF-PANet and GSconv is abbreviated as CTG-YOLO.

The results of the ablation experiment (Table 4) validate the effectiveness of the improved modules in terms of accuracy, lightweight design, and computational efficiency from the perspective of five dimensions, such as AP and mAP.

CBY Module: C-YOLO introduces the CBY parallel structure as the backbone module. The number of parameters increases to 13.227 M, and the computational complexity rises to 30.463 G. The mAP increases to 73.36%, with a detection speed of 118 FPS (2 FPS lower than the baseline), achieving a preliminary balance between accuracy and efficiency.

TFF Module: T-YOLO incorporates the TFF module in the neck network. The computational complexity increases to 33.092 G, and the detection speed drops to 115 FPS. However, the AP for Cr improves to 41.51%, and the mAP reaches 74.07%, demonstrating the value of multi-scale feature fusion.

GSConv Module: G-YOLO replaces the standard convolution in the prediction head with the lightweight GSConv module. The mAP rises to 74.60%, the number of parameters decreases to 9.196 M, and the computational complexity drops to 21.294 G. The detection speed increases to 132 FPS (12 FPS higher than the baseline), showcasing its advantage in terms of lightweight design.

Multiple-Module Combination: CT-YOLO integrates both CBY and TFF modules, increasing the mAP to 75.97%, highlighting the combined advantages of the two modules. TG-YOLO combines the TFF module and GSConv, achieving no loss in accuracy and lower computational cost than the baseline. CTG-YOLO integrates all three modules, with 13.667 M parameters and a computational complexity of 27.216 G (lower than the baseline). The detection speed is 122 FPS (slightly higher than the baseline), and the mAP reaches 76.55%. Each module works effectively on its own, and although some optimizations may “cancel out” when combined, the overall effect is significant.

### 4.6. Class-Wise Error Analysis

To quantitatively reveal the error distribution and category confusion characteristics of the CTG-YOLO model in steel surface defect detection, this section systematically analyzes the sources of detection errors by combining the precision, recall, and F1 score for each defect category based on the general industrial defect detection matching standard (IoU threshold of 0.5). The results are shown in Table 5 and Table 6.

Specifically, precision reflects the proportion of samples predicted by the model as a specific defect category that are truly of that category, demonstrating the model’s ability to suppress false positives (FPs). Recall reflects the proportion of samples that truly belong to a specific defect category and are correctly detected by the model, demonstrating the model’s ability to suppress false negatives (FNs). The F1 score, as the harmonic mean of the two, comprehensively measures the model’s detection accuracy for that defect category. Through these metrics, the model’s detection performance for each defect category can be further quantified, clarifying its main error types.

#### 4.6.1. Classification Performance Metrics for the NEU-DET Dataset

Based on the data in Table 5, a systematic analysis of the model’s detection performance and core issues for each defect category can be performed.

Crazing (Cr, the most challenging category) exhibits extreme characteristics of high precision and extremely low recall; its precision of 72.73% indicates that the model has strong classification accuracy for the detection of Cr cracks (the proportion of misclassification as other categories is low). However, the recall is only 11.11%, meaning that only about 33 out of 300 Cr samples were successfully detected, with nearly 90% of the samples being missed due to low contrast, small size, and complex texture (dominated by false negatives, FNs). This directly results in an F1 score of only 19.00%, which becomes a critical bottleneck limiting the overall performance of the model.

Scratches (Sc, the best detected category): With a recall of 94.12%, precision of 87.27%, and F1 score of 91.00%, this category has the best performance for all metrics. This is because SC scratches often present clear linear textures, with high contrast against the background and regular shapes. The model’s CBY parallel module and TFF-PANet can effectively capture its features, leading to extremely stable detection.

Rolled in scale (Rs, moderate detection category) achieves a precision of 82.86%, recall of 39.19%, and F1 score of 53.00%, making it the worst performing category, except for Cr cracks. The core issue lies in the low recall rate. Rs defects are often irregularly distributed iron scale imprints, with some areas showing high similarity to the background texture, causing a large number of samples to be missed. Meanwhile, precision is lower than that of the Pa, Ps, and In categories, indicating some confusion with the scratches category. The combination of these two factors leads to poor overall detection performance.

In summary, the model’s detection performance on the NEU-DET dataset shows significant differentiation: The categories of scratches, patches, and pitted surfaces perform excellently, whereas inclusion shows moderate performance, while crazing and rolled-in-scale defects are the main weaknesses. The extremely low recall rate of crazing is the core issue and requires targeted improvements by enhancing the low-contrast defect feature signals and optimizing the multi-scale feature fusion strategy. Rolled-in-scale defects, on the other hand, require a focus on improving recall while suppressing category confusion errors.

#### 4.6.2. Classification Performance Metrics for the GC10-DET Dataset

The GC10-DET dataset covers 10 common steel surface defects in industrial scenarios, with samples that are more representative of the complexity in real production environments. The classification performance metrics are shown in Table 6.

High-performance detection categories (F1 ≥ 80%) include Pu (punching), Wl (weld seam), Cg (crescent-shaped gaps), and Ws (water stains). The key advantage of these defects is their distinct morphological features and high contrast with the background. Among them, Pu defects achieve 100% precision, 93.94% recall, and an F1 score of 97%, performing the best. Punching defects are mostly regular circular or square holes with clear boundaries and unique features. The model’s CBY parallel module can fully capture their contour information, with almost no missed detection or false positives. Wl (weld seam) defects feature long, continuous stripes, and Cg (crescent-shaped gap) defects have a unique contour, so both are easily distinguishable. Although Cg’s precision is only 76.92%, its high recall rate of 95.24% means very few missed detections, meeting the industrial requirement of “prioritizing the prevention of missed key defects”.

Moderate detection categories (60% ≤ F1 < 80%) include Os (oil stains), Ss (silk marks), and Wf (waist creases). These defects share the characteristic of having somewhat dispersed features: Os (oil stains) are mostly irregular and sheet-like, with some areas having small differences in grayscale from the background, leading to a recall rate of only 56.60%. Ss (silk marks) are fine, scattered defects with weak feature signals, resulting in a recall rate of 53.75%. Wf (waist creases) have 100% precision (no false positives), but due to some shallow creases overlapping with the steel rolling texture, their recall rate is 63.64%, with an overall F1 score of 78%.

Low-performance detection categories (F1 < 60%) include Rp (roll pits), Cr (creases), and In (inclusions), which represent the core weaknesses in the model’s detection and all exhibit “low recall rate” characteristics. For Rp, a precision of 100% means that once the model classifies a defect as a roll pit, it is completely accurate, with no category confusion, but its recall rate is only 20%. This means only about 46 out of 229 samples are successfully detected, with nearly 80% of the samples misclassified as background due to the “shallow depth of roll pits and high similarity to background textures,” making it one of the most challenging defect types in industrial scenarios. For In, we report a precision of 60%, recall rate of 13.04%, and an F1 score of 21%, all of which are the lowest. Inclusion defects are often small, scattered impurity points with very low contrast and prone to being confused with surface noise on the steel surface, resulting in significant missed detections and some false positives, leading to the worst detection performance. For Cr, the recall rate of 14.29% is similar to that for In, with only about 10% of samples detected. This is mainly because some creases are “latent creases,” with no obvious convex or concave features or grayscale differences, and their feature signals are weak, making them difficult to capture using the feature extraction module.

In summary, the model’s detection performance on the GC10-DET dataset exhibits significant “category dependence”; defects with regular shapes and high contrast are detected effectively, while smaller, low-contrast defects with high similarity to the background perform poorly. Specifically, the “high precision, low recall” issue with Rp requires enhancement of low-contrast defect feature signals, while In’s “low precision, low recall” requires optimization of both category-specific feature extraction and noise suppression capabilities to meet the industrial requirements for defect detection.

#### 4.6.3. Quantitative Evaluation Based on the Confusion Matrix

**Figure 11:**
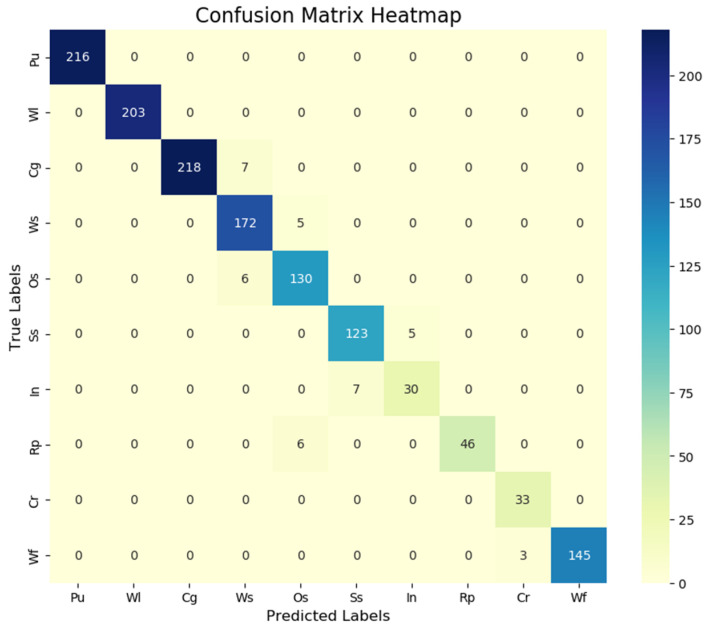
Confusion matrix heatmap of CTG-YOLO on the GC10-DET dataset.

To quantitatively reveal the category confusion characteristics of the CTG-YOLO model in steel surface defect detection, this section adds a confusion matrix-based analysis on top of the original precision, recall, and F1 score. Using the commonly adopted IoU = 0.5 matching threshold in industrial defect detection, a confusion matrix for the CTG-YOLO model on the GC10-DET dataset is constructed (Figure 11). The rows of the matrix represent the true defect categories, while the columns represent the categories predicted by the model. The values in the matrix correspond to the number of matched samples for each category, presenting the degree of confusion between categories and the background misclassification situation.

The analysis results indicate that perforation (Pu) and weld seam (Wl) defects exhibit good distinguishability, with diagonal percentages of 93.94% and 88.46%, respectively, and no obvious misclassifications across categories. This is due to the regular morphological characteristics of these two types of defects, which significantly differ from other defect categories, allowing the model to accurately identify them.

In the main confusion pairs, there is a significant mix-up between pitting (Rp) and oil spots (Os), with 2.62% of the samples in Rp being misclassified as Os. This is due to the similarity in the grayscale features of the dark area at the bottom of pitting and oil-spot defects. The confusion between inclusions (In) and streaks (Ss) is more prominent, with 3.06% of the samples in In being misclassified as Ss, as both are fine, dispersed defects that are difficult to distinguish under low-contrast conditions. There is also confusion between crescent-shaped cracks (Cg) and water spots (Ws), with 3.06% of the samples in Cg being misclassified as Ws. This is due to the similarity between the blurry curved edges of crescent-shaped cracks and the texture of water spots.

A particularly notable issue is the background misclassification problem: The background misclassification rates for inclusions (In), pitting (Rp), and wrinkles (Cr) are as high as 86.90%, 79.91%, and 85.59%, respectively, all exceeding 75%, while the inter-category confusion rate is below 3.06%. Further data analysis confirms that the core bottleneck in detecting these types of defects lies in insufficient feature extraction capability. The model primarily faces the issue of missed detection rather than inter-category misclassification.

#### 4.6.4. Quantitative Counts of TP/FP/FN for All Categories

This section presents the TP, FP, and FN counts of the CTG-YOLO model for each category on the NEU-DET dataset, specifying the number of correctly detected instances and the scale of errors for each defect type. The results are shown in Table 7.

Table 8 shows the FP/FN breakdown by category for the CTG-YOLO model on this dataset, along with key evaluation metrics. The proportion of failure cases and representative examples are analyzed as follows. Crazing (Cr) exhibits a characteristic of missed detection: there are only 33 TP instances, while the number of FNs reaches 267, resulting in a missed detection rate of 89.00%. The features of this defect are highly fused with the steel rolling background, making it difficult for the CBY parallel module to capture faint feature signals. Nearly 90% of the samples are misclassified as background. It is worth noting that there are only three misclassifications and four background FPs, indicating that the model’s ability to distinguish the Cr category and differentiate from the background is relatively strong. The core bottleneck lies in insufficient feature capture, leading to missed detections, rather than category confusion or background misclassification.

Scratches (Sc) exhibit prominent background misclassification: There are 282 TP instances, with a recall rate of 94.00% and a missed detection rate of only 6.00%. However, there are 41 FP instances, of which 31 are background FPs. As a high-contrast linear defect, the model has strong ability to capture this type of feature, resulting in a very low missed detection rate. However, the normal rolling texture on the steel surface resembles the shape of Sc defects, leading to a significant number of background misclassifications. The core issue lies in the insufficient ability to differentiate between defects and background linear textures.

In summary, the detection performance of the CTG-YOLO model on the NEU-DET dataset shows significant differentiation. Scratches have the highest detection rate but suffer from severe background misclassification. Patches, pitting, and inclusions show stable performance, while cracks and pressed iron oxide scale are the main issues. The core problem with cracks lies in insufficient feature capture, leading to missed detections, while pressed iron oxide scale faces the dual challenge of missed detections and category confusion. Future optimizations should focus on enhancing feature extraction capabilities and improving the model’s ability to differentiate background textures.

## 5. Conclusions

This paper proposes the CTG-YOLO model for steel surface defect detection, which further improves detection accuracy in industrial environments. First, to address the issue of insufficient feature interaction for complex defect shapes, we designed the ConvNext-C2f parallel module to expand the receptive field, enhance feature interaction, and improve multi-scale feature extraction. Second, to overcome the problem of low feature extraction capability in the neck network due to the lack of multi-scale fusion, we introduced the TFF-PANet neck structure. This aggregates multi-scale features from PANet to capture richer semantic information and enhance feature reuse, reducing information loss. Lastly, we designed the GSConv detection head to maximize inter-channel information connectivity, reduce model size, and improve performance. Extensive experiments show that the improved CTG-YOLO model outperforms other methods in terms of detection accuracy and versatility. Notably, the design of these modules follows the core principles of the base network, making them adaptable to other deep learning models for improved multi-scale detection performance, showing great potential in steel defect detection tasks. Moreover, the optimization methods for the detection of low-contrast, small defects (such as multi-scale feature fusion, channel information retention, and lightweight feature extraction) provide new research ideas for similar detection challenges in industrial quality inspection, filling the technical gaps in current methods and offering significant theoretical and practical value.

Despite the overall strong performance of the improved model, its detection accuracy for blurred, complex, and subtle defects (e.g., cracks and rolling defects) remains relatively low, suggesting that CTG-YOLO’s performance still requires further refinement. This study also has some limitations. Specifically, the current analysis lacks detailed contrast metrics for low-contrast defect detection. Future research could focus on quantifying the contrast values of such defects and conducting controlled ablation studies to more effectively explore the impact of low-contrast defects on detection accuracy, providing more precise data for model optimization. Future work should also prioritize the integration of shallow feature enhancement and contrast enhancement techniques in image preprocessing, continuously optimizing model performance while addressing the practical demands of industrial deployment, thereby further improving CTG-YOLO’s ability to detect defects in complex backgrounds.

## Figures and Tables

**Table 1:** Configuration of the experimental environment.

Setup of the Environment	Version and Model
Operating System	Windows 11
CPU	12 vCPU Intel(R) Xeon(R) Silver 4214R CPU @ 2.40 GHz
GPU	RTX 3080 Ti (12 GB)
Deep Learning Framework	PyTorch 1.7.0
Accelerator	CUDA 11.0

**Table 2:** Average accuracy evaluation parameter.

	The Actual Situation Is Positive	The Actual Situation Is Negative
The test result is positive	True Positive (TP)	False Positive (FP)
The test result is negative	False Negative (FN)	True Negative (TN)

**Table 3:** Detection results of different models on the NEU-DET dataset.

Model	AP/%						mAP/%	Params/M	FLOPs/G	FPS
	Cr	In	Pa	PS	RS	SC				
Faster-RCNN	43.7	63.7	83.4	74.9	66.2	89.9	70.3	134.62	402.02	20.7
SSD	39.6	58.5	72.4	71.8	61.8	70.9	62.5	26.43	145.3	41
EfficientDet [29]	45.9	62.0	83.5	85.6	70.7	73.1	70.1	20.7	42.1	11.9
YOLOv3-tiny	44.3	74.3	91.6	69.1	65.7	66.9	68.6	8.6	12.9	117
YOLOv4 [30]	34.7	76.8	87.4	76.5	50.3	81.6	67.8	35.1	37.2	45
YOLOv7-tiny [31]	39	86.6	79.2	66.8	55.9	71.3	66.4	6	13.8	70
YOLOv8s	35.58	79.45	90.73	87.97	54.17	89.11	72.83	11.167	28.817	120
YOLOv9s	42.8	81.6	89.7	77.1	67.8	93.3	75.5	20.3	38.7	31
YOLOv10s [32]	44.5	76.5	85.9	77.2	60.4	89.0	72.3	7.2		71
RetinaNet [33]	32.2	75.6	75.4	62.0	82.0	83.1	68.4	32.1	30	87
FCOS [34]	36.3	89.3	84.0	61.0	81.9	90.7	73.9	30.7	50	72
CTG-YOLO	42.09	84.67	92.02	90.35	56.38	93.81	76.55	13.667	27.216	122

**Table 4:** Detection results of different models on the GC10-DET dataset.

Model	Pu (%)	WI (%)	Cg (%)	Ws (%)	Os (%)	Ss (%)	In (%)	Rp (%)	Cr (%)	Wf (%)	mAP (%)
Faster-RCNN	94.4	96.2	80.3	72.1	40.7	38.8	11.5	19.9	40.3	22.6	51.7
SSD [36]	91.6	74.4	92.8	81.5	43.0	58.0	1.0	12.5	42.3	33.3	53.0
EfficientDet	96.8	87.3	93.6	71.0	62.8	63.6	14.1	28.0	8.3	88.2	61.4
YOLOv3-tiny	96.0	90.1	95.0	87.6	60.2	54.6	39.4	14.1	15.2	86.1	60.2
YOLOv8	97.9	78.4	96.0	87.8	80.2	67.1	15.9	23.3	37.0	84.2	66.8
YOLOv9 [37]	92.5	79.8	98.9	79.6	83.0	66.8	19.7	29.1	56.1	80.8	68.6
FCOS [34]	96.0	58.0	92.6	74.0	61.5	61.8	21.3	35.7	25.1	84.2	61.0
FPDNet [32]	97.1	94.7	94.2	72.5	63.9	40.2	35.3	45.0	40.9	84.1	66.8
Liu’s [35]	97.7	95.2	92.5	75.2	67.0	61.1	37.6	48.8	31.2	84.5	69.1
CTG-YOLO	93.94	93.88	88.53	79.56	68.67	64.43	45.79	23.75	52.38	88.48	69.94

**Table 5:** Ablation experiment results of CTG-YOLO on the NEU-DET dataset.

Methods	mAP/%	AP/%						Params/M	FLOPs/G	FPS
		Cr	In	Pa	PS	RS	SC			
Baseline	72.83	35.58	79.45	90.73	87.97	54.17	89.11	11.167	28.817	120
C-YOLO	73.36	34.52	78.16	91.77	88.25	54.90	92.57	13.227	30.463	118
T-YOLO	74.07	41.51	82.52	91.11	83.56	54.26	91.52	13.576	33.092	115
G-YOLO	74.6	32.87	81.10	94.78	86.49	56.12	96.37	9.196	21.294	132
CT-YOLO	75.97	42.43	83.03	95.13	87.67	56.05	91.50	15.637	34.739	112
CG-YOLO	74.86	32.88	82.72	95.88	86.96	55.69	95.05	11.257	22.940	128
TG-YOLO	74.67	38.39	79.75	93.22	87.57	55.95	93.19	11.606	25.569	125
CTG-YOLO	76.55	42.09	84.67	92.02	90.35	56.38	93.81	13.667	27.216	122

**Table 6:** Precision, recall, and F1 score for each defect category in the NEU-DET dataset.

Defect Class	Total Samples	Precision/%	Recall/%	F1 Score/%
Cr	300	72.73	11.11	19
In	300	92.31	70.59	80
Pa	300	97.10	79.76	88
Ps	300	94.20	77.38	85
Rs	300	82.86	39.19	53
SC	300	87.27	94.12	91

**Table 7:** Precision, recall, and F1 score for each defect category in the GC10-DET dataset.

Defect Class	Total Samples	Precision/%	Recall/%	F1 Score/%
Pu	229	100	93.94	97
Wl	229	97.87	88.46	93
Cg	229	76.92	95.24	85
Ws	229	88.89	75	81
Os	229	85.71	56.60	68
Ss	229	78.18	53.75	64
In	229	60	13.04	21
Rp	229	100	20	33
Cr	229	66.67	14.29	24
Wf	229	100	63.64	78

**Table 8:** TP/FP/FN counts and error-type breakdown for each defect category.

Defect Class	TP	FP	FN	Misclassification	Localization	Background FP
Cr	33	12	267	3 (Misclassified as Rs)	5	4
In	212	18	88	5 (Misclassified as Pa)	7	6
Pa	239	7	61	2 (Misclassified as Ps)	3	2
Ps	232	14	68	4 (Misclassified as Pa)	6	4
Rs	118	24	182	8 (Misclassified as Sc)	10	6
Sc	282	41	18	1 (Misclassified as Rs)	9	31

## Data Availability

The data presented in this study are openly available in CTG-YOLO https://github.com/mystery-you/CTG-YOLO (accessed on 16 March 2026).
